# Computerized Block Games for Automated Cognitive Assessment: Development and Evaluation Study

**DOI:** 10.2196/40931

**Published:** 2023-05-16

**Authors:** Xiangyi Cheng, Grover C Gilmore, Alan J Lerner, Kiju Lee

**Affiliations:** 1 Dr Carl D and H Jane Clay Department of Mechanical Engineering TJ Smull College of Engineering Ohio Northern University Ada, OH United States; 2 Department of Psychological Sciences Case Western Reserve University Cleveland, OH United States; 3 Department of Neurology Case Western Reserve University Cleveland, OH United States; 4 Department of Engineering Technology and Industrial Distribution Texas A&M University College Station, TX United States; 5 J Mike Walker '66 Department of Mechanical Engineering Texas A&M University College Station, TX United States

**Keywords:** cognitive assessment, computerized block games, adaptive serious games, computerized cognitive assessment

## Abstract

**Background:**

Cognitive assessment using tangible objects can measure fine motor and hand-eye coordination skills along with other cognitive domains. Administering such tests is often expensive, labor-intensive, and error prone owing to manual recording and potential subjectivity. Automating the administration and scoring processes can address these difficulties while reducing time and cost. e-Cube is a new vision-based, computerized cognitive assessment tool that integrates computational measures of play complexity and item generators to enable automated and adaptive testing. The e-Cube games use a set of cubes, and the system tracks the movements and locations of these cubes as manipulated by the player.

**Objective:**

The primary objectives of the study were to validate the play complexity measures that form the basis of developing the adaptive assessment system and evaluate the preliminary utility and usability of the e-Cube system as an automated cognitive assessment tool.

**Methods:**

This study used 6 e-Cube games, namely, Assembly, Shape-Matching, Sequence-Memory, Spatial-Memory, Path-Tracking, and Maze, each targeting different cognitive domains. In total, 2 versions of the games, the fixed version with predetermined sets of items and the adaptive version using the autonomous item generators, were prepared for comparative evaluation. Enrolled participants (N=80; aged 18-60 years) were divided into 2 groups: 48% (38/80) of the participants in the *fixed group* and 52% (42/80) in the *adaptive group*. Each was administered the 6 e-Cube games; 3 subtests of the Wechsler Adult Intelligence Scale, Fourth Edition (WAIS-IV; Block Design, Digit Span, and Matrix Reasoning); and the System Usability Scale (SUS). Statistical analyses at the 95% significance level were applied.

**Results:**

The play complexity values were correlated with the performance indicators (ie, correctness and completion time). The adaptive e-Cube games were correlated with the WAIS-IV subtests (*r*=0.49, 95% CI 0.21-0.70; *P*<.001 for Assembly and Block Design; *r*=0.34, 95% CI 0.03-0.59; *P*=.03 for Shape-Matching and Matrix Reasoning; *r*=0.51, 95% CI 0.24-0.72; *P*<.001 for Spatial-Memory and Digit Span; *r*=0.45, 95% CI 0.16-0.67; *P*=.003 for Path-Tracking and Block Design; and *r*=0.45, 95% CI 0.16-0.67; *P*=.003 for Path-Tracking and Matrix Reasoning). The fixed version showed weaker correlations with the WAIS-IV subtests. The e-Cube system showed a low false detection rate (6/5990, 0.1%) and was determined to be usable, with an average SUS score of 86.01 (SD 8.75).

**Conclusions:**

The correlations between the play complexity values and performance indicators supported the validity of the play complexity measures. Correlations between the adaptive e-Cube games and the WAIS-IV subtests demonstrated the potential utility of the e-Cube games for cognitive assessment, but a further validation study is needed to confirm this. The low false detection rate and high SUS scores indicated that e-Cube is technically reliable and usable.

## Introduction

### Background

Cognitive assessment aims to measure multiple domains of cognition, including visuospatial abilities, working memory, language, attention, executive function, fine motor skills, and orientation [1]. One’s cognitive abilities affect learning outcomes, physical and mental health, social behavior, and interaction with the environment [2-4]. Identifying impairment in any of these domains, diagnosing the cause, specifying the severity, and tracking the progression of the symptoms are the common purposes of cognitive assessment in clinical settings [5]. This paper presents an innovative technology called *e-Cube* for adaptive, automated cognitive testing and reports the evaluation results in terms of preliminary utility and usability.

There are standardized instruments widely used for cognitive assessment. The Wechsler Adult Intelligence Scale (WAIS) has been broadly adopted in clinical, research, and educational settings and is often referred to as a *gold* standard [6]. The WAIS Fourth Edition (WAIS-IV) is normed for the ages of 16 to 90 years. It comprehensively assesses cognitive abilities using 15 subtests that target various cognitive domains [7]. This instrument is administered and scored by a qualified psychologist, taking approximately 60 to 90 minutes. This process is labor-intensive and costly [8]. The Stanford-Binet Intelligence Scales, Fifth Edition, is another standardized instrument commonly used in both clinical and research settings [9]. Several WAIS and Stanford-Binet Intelligence Scales subtests rely heavily on a person’s verbal skills and, therefore, show limitations when administered using a non–native language version [10]. There are also nonverbal instruments aiming to eliminate cultural and language biases in the assessment. For example, the Raven Progressive Matrices consist of 60 items measuring the basic cognitive functioning of individuals, each of which is a visual geometric design with a missing piece [11,12].

The advancements in digital technologies have enabled researchers to explore computer-based methods for cognitive assessment. Computer-based methods can reduce the administrative burden, automate the scoring process, reduce cheating, and standardize test conditions once successfully validated [13]. A straightforward application is to convert a paper-and-pencil test into a computerized version while retaining the contents and formats. Q-interactive is a digital system initially developed for the WAIS-IV that uses 2 iPads, one for the administrator and the other for the test taker [14]. This digital version reduces labor-intensity but takes approximately the same time for a trained professional. Moreover, it can only automate some types of tests. In particular, one of the subtests, Block Design (BD), requires the examinee to assemble physical blocks to match the top surface with a given image displayed on an iPad. The administrator then has to check the correctness and input the results manually. In addition to the computerization of existing instruments, an increasing body of research has adopted the concept of computer- or tablet-based *serious games* to make the experience more engaging [15-17]. Some serious games use dynamic difficulty adjustment to achieve adaptive testing by tuning item difficulty autonomously [18,19]. However, most of the previously developed games for cognitive assessment do not include measurements of fine motor and hand-eye coordination skills.

### Cognitive Assessment Using Tangible Objects

Cognitive assessment sometimes uses tangible objects to measure one’s cognitive skills together with fine motor and hand-eye coordination skills. These skills are closely linked to many neurological diseases and brain injuries [20]. Existing research also suggests that the deterioration of fine motor control and coordination characterizes sensorimotor deficiencies in mild cognitive impairment and Alzheimer disease [15,21-23]. The BD subtest in the WAIS [5] and the Kohs Block Design test [24] use a set of cubes and require an examinee to place and assemble the top surfaces of the blocks to match the given image. Unlike the simple multiple-choice questions used in many other assessment instruments, the administrator has to inspect the correctness of the block manipulation visually while timing in these tests. This is labor-intensive and error prone owing to manual recording and subjectivity, possibly affecting the assessment results.

Automating the assessment using physical objects also involves additional challenges and requires technological innovations beyond what is expected for computerized tests. For example, a platform called ETAN supports the use of tangible user interfaces and physical objects for evaluating visuospatial cognition by implementing the Baking Tray Task [25]. Cognitive Cubes were designed to assess spatial and constructive abilities by asking users to build 3D shapes with the cubes. A pilot study involving 16 participants demonstrated that the Cognitive Cubes were sensitive to differences in cognitive ability [20].

SIG-Blocks and TAG-Game, developed for the automated assessment of cognitive and fine motor skills, were the previous research of this work [26-28]. Each SIG-Block, covered with simple black-and-white geometric shapes, can sense physical motions applied to it, detect adjacent blocks, and send sensor data to a local host computer in real time. TAG-Games are computerized games that use SIG-Blocks as a means of game control. In total, 3 types of TAG-Games, namely, Assembly, Shape-Matching, and Memory, were designed and tested. These games are all nonverbal and require hand manipulation of physical blocks. The TAG-Game technology is one of the few systems that can automate the administration and data collection of tasks involving physical object manipulation. However, despite its demonstrated potential, several challenges were identified in our previous research. Specifically, hardware costs, occasional technical failure, and high maintenance make the system unsuitable for broad and long-term adoption and use.

### e-Cube Games for Automated Assessment of Fine Motor and Cognitive Skills

e-Cube is our latest technical innovation that converts the original TAG-Game system into a computer vision-based system using a set of plastic cubes and a webcam. The e-Cube system reduces the device cost from US $1500 to approximately US $50 (excluding a computing device needed for any computerized assessment), decreases potential technical errors, and nearly eliminates the maintenance burden. The entire system is fully autonomous and easy to use. In addition to these benefits, a new *adaptive* test environment was established based on the embedded algorithms for measuring play complexity and generating adaptive test items autonomously. These features enable personalized assessment based on an individual’s real-time performance. e-Cube consists of 6 types of games: (1) Assembly, (2) Shape-Matching, (3) Sequence-Memory, (4) Spatial-Memory, (5) Path-Tracking, and (6) Maze. The first 3 were directly adopted and converted from TAG-Games, and the other 3 were newly created. New computational measures of play complexity were defined and implemented for each game.

The evaluation focused on testing 2 objectives. *Objective 1* was to validate the proposed play complexity measures that form the algorithmic basis of the adaptive games. Correlation analyses were performed between the developed play complexity measures and 2 performance indicators, mean correctness and mean completion time. *Objective 2* was to understand the preliminary utility and usability of the e-Cube system as an automated cognitive assessment tool. The non–age-corrected raw scores of 3 WAIS-IV subtests—BD, Digit Span (DS), and Matrix Reasoning (MR)—were adopted to compare their results with the e-Cube game scores. The WAIS-IV is a well-established instrument, and the 3 selected subtests measure the target cognitive domains of the e-Cube games. Specifically, the Assembly game was conjected to be related to BD as both require the assembly of block surfaces to match a given pattern. The Shape-Matching game requires the participant to find a shape that completes a pattern, so it was expected to tap the same cognitive abilities as the MR subtest. Sequence-Memory and Spatial-Memory were expected to be related to DS as they all target working memory skills. The remaining games, Path-Tracking and Maze, are timed games asking participants to give the shortest trajectory by reasoning, so they were both hypothesized to show a relationship with BD and MR. The *hypothesized relationships* between the e-Cube games and the WAIS subtests are summarized in Table 1. The false detection rate of the system determines whether it produces reliable and accurate data. Usability was evaluated by administering the System Usability Scale (SUS) to all participants upon the completion of the assessment session. The SUS is a 10-item questionnaire measuring usability with high validity and reliability and, thus, used as a measure of perceived usability [29-31].

**Table 1:** The 6 e-Cube games with their associated task descriptions and the expected associations with the Wechsler Adult Intelligence Scale, Fourth Edition (WAIS-IV), subtests (Block Design [BD], Digit Span [DS], and Matrix Reasoning [MR]).

e-Cube game	Task	WAIS-IV		
		BD	DS	MR
Assembly	Assemble multiple cubes to match the top assembly configuration	✓		
Shape-Matching	Manipulate 1 cube to complete the pattern with 1 missing piece			✓
Sequence-Memory	Memorize a sequence of geometric shapes and reconstruct it using 1 cube		✓	
Spatial-Memory	Memorize a spatial assembly of geometric shapes and reconstruct it using cubes		✓	
Path-Tracking	Trace a connected path between 2 blue dots using a single cube	✓		✓
Maze	Navigate through a maze to reach a goal point from a starting point using a single cube	✓		✓

## Methods

### e-Cube Games

#### System Overview

**Figure 1:**
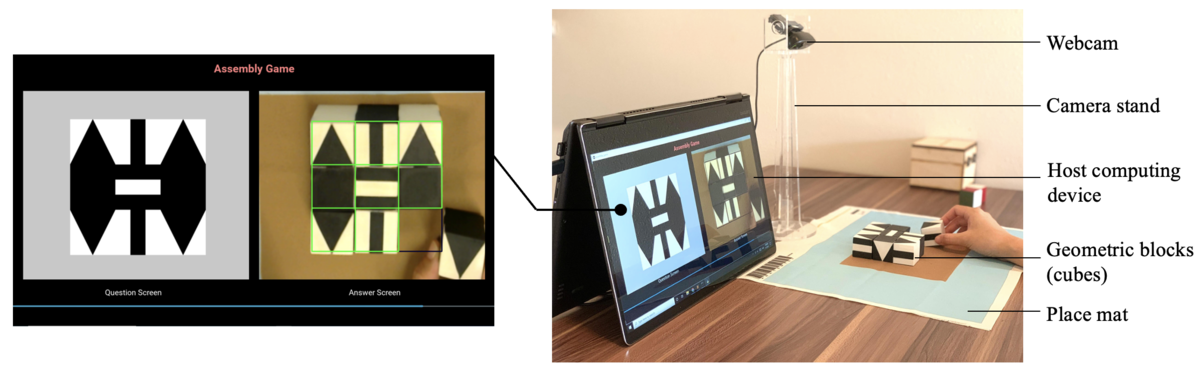
The hardware of e-Cube, consisting of the cubes, a webcam with a stand, a place mat, and a host computing device running the Assembly game.

**Figure 2:**
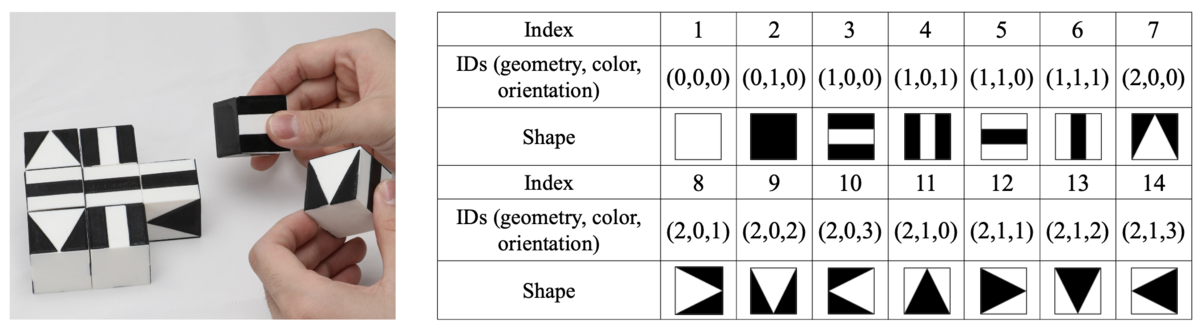
A total of 9 geometric cubes and 14 distinctive surface shapes with their IDs formed by rotating the images on the 6 surfaces of a cube by 0°, 90°, 180°, and 270°.

The e-Cube system consists of a set of 9 cubes with 1.2-inch–length edges, a place mat with a brown rectangular region in the center, a computing device with a display, and a webcam with a custom-designed stand (Figure 1). The cube’s 6 faces are distinctive black-and-white geometric shapes, including squares, strips, and triangles representing 4-, 2-, and 1-fold rotational symmetry (Figure 2). The cubes preserve the same design as the SIG-Blocks [28]. When the system turns on, the camera automatically detects the corners of the brown rectangular area on the place mat. This area is called the *play area,* where the cubes are expected to be placed and manipulated. The laptop with the connected webcam displays the cubes in the play area after perspective transformation projecting the original camera view onto a 2D plane and tracks their movements in real time [32]. This autonomous transformation offers flexibility in the camera location.

The e-Cube system requires the accurate identification of the top-surface images of the cubes. Individual box-shaped regions are assigned for placing the cubes (Figure 1), wherein the geometric shape and orientation detection algorithm is executed. The embedded algorithm first detects the black-and-white regions within each box to check whether a cube exists. It then identifies a polygon using the Ramer-Douglas-Peucker algorithm [33]. Finally, the specific shape and orientation of the detected polygon are determined. This simple strategy makes the system robust and reliable under different illumination conditions and limited computing capabilities. The e-Cube system can run reliably on a relatively low-end computing device, such as Intel Core i5-7200U (2.5 GHz, 3 M cache, dual core, and 4 threads).

#### Game Design

We developed 6 e-Cube games: Assembly, Shape-Matching, Sequence-Memory, Spatial-Memory, Path-Tracking, and Maze. We directly converted the 3 TAG-Games (Assembly, Shape-Matching, and Memory) into the vision-based e-Cube versions (Assembly, Shape-Matching, and Sequence-Memory) [26]. Spatial-Memory, Path-Tracking, and Maze were newly added.

**Figure 3:**
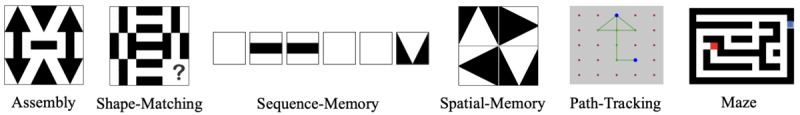
Sample items for the 6 games.

Table 1 presents the tasks associated with each game, and Figure 3 shows an example item for each e-Cube game. Assembly asks the player to match the given assembly figure displayed on the screen using 4 or 9 cubes, similar to the BD subtest of the WAIS-IV. Shape-Matching involves items with assembly patterns, each missing 1 piece, and the player completes the pattern using a single cube. Sequence-Memory and Spatial-Memory require the player to memorize a sequence or an assembly of geometric shapes. In Sequence-Memory, each shape is displayed for 1 second and then disappears. In Spatial-Memory, an assembly pattern of 2, 3, or 4 geometric shapes is displayed for 5 seconds. The items in the Spatial-Memory game are similar to those in Assembly, whereas visible outlines around individual shapes are added, as shown in Figure 3, to assist perceptual segmentation of the pattern [34]. Path-Tracking and Maze use only 1 cube with its white square facing up. In these 2 games, the vision algorithm detects the center of the white square and tracks it continuously on the screen; no assigned box-shaped regions are shown on the screen. Path-Tracking displays a green connected path between 2 blue dots, and the player must trace the path by moving the cube from one blue dot to the other on a 5 × 5 grid via the shortest path. The Maze game asks the player to find the shortest path of mazes shown on the screen by moving a cube from the start (blue) to the end (red).

#### Computational Measures of Play Complexity

##### Overview

**Figure 4:**
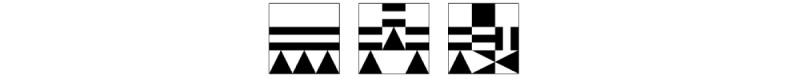
Items formed by the same geometric shapes but with different play complexity (with identical compositional complexity but different configurational complexity).

The e-Cube system aims to dynamically adapt to individual differences in cognitive skills by generating test items autonomously based on one’s real-time performance. To do so, a computational method to measure the difficulty of each item is required. The previously defined measures of play complexity presented in the studies by Lee et al [26,35] and Jeong et al [28] were highly correlated with the participants’ performances measured using completion time or accuracy. These measures captured the complexities associated with individual geometric shapes without considering the spatial complexity of the assembly patterns. For example, the 3 assembly patterns shown in Figure 4 had the same complexity value using the previously defined measures. As our previous study used a handcrafted set of items, we could select the items where their difficulties could be clearly differentiated using the previously defined measures. However, for generating adaptive test items, the complexity measures must capture the difficulties associated with both the individual shapes and the assembly patterns.

To address this limitation, we defined new complexity measures for the 6 e-Cube games. Two mathematical concepts were applied: (1) the Shannon entropy and (2) the gray-level co-occurrence matrix (GLCM). The Shannon entropy measures the uncertainty, randomness, or disorder existing in the data [36] and is calculated as



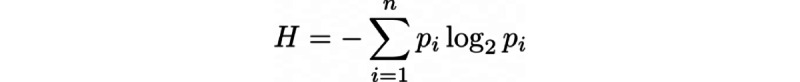



where *p_i_* is the probability of the *i*th event. When the probabilities are evenly distributed, the Shannon entropy is calculated as *H*=log_2_*n*. The GLCM was originally proposed to classify image texture in grayscale [37,38]. For an image with an *m* × *n* dimension and *L* gray level, the GLCM of the image (*f*) is defined as an *L* × *L* square matrix such that



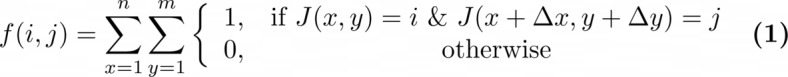



where Δ*x* and Δ*y* are typically defined as the horizontal, vertical, or diagonal position differences between the 2 adjacent pixels [37]. Horizontally adjacent pixels can be paired along 0° or 180°; vertically adjacent pixels can be paired along 90° or 270°; and diagonally adjacent pixels can be paired along 45°, 135°, 225°, or 315°. On the basis of the Shannon entropy and the GLCM, the computational measures of play complexity for the 6 e-Cube games are defined in the following sections.

##### Play Complexities of Assembly, Sequence-Memory, Spatial-Memory, and Shape-Matching

The play complexity of the items in Assembly, Sequence-Memory, and Spatial-Memory is computed using



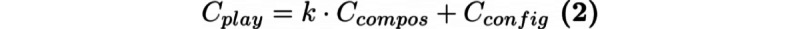



where *C_compos_* represents the compositional complexity associated with individual shapes (ie, the number of shapes and their rotational symmetry), *C_config_* captures the configurational complexity associated with the orientation and color differences among the shapes in the way that they are arranged, and *k* is a sigmoid function defined as



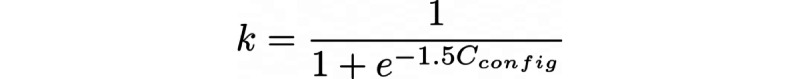



If *C_config_* is small, a small *k* leads to a lower impact of *C_compos_* on *C_play_*. For example, if an item is formed only by identical triangles (large *C_compos_* and small *C_config_*), *C_play_* will still be small owing to *k*.

The Shannon entropy forms the basis of *C_compos_* such that



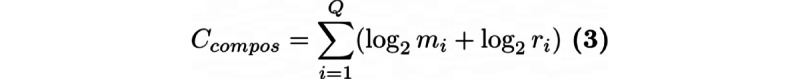



where *Q* is the total number of shapes in the item, *m*_i_ is the number of available distinctive shapes among the 6 faces of a cube (*m*_i_=6 if all faces of a cube are different), and *r*_i_ is the number of distinctive orientations obtained by rotating this shape 90° (*r*_i_=1 for squares, 2 for strips, or 4 for triangles). The 3 images in Figure 4 have the same *C_compos_* value.

**Figure 5:**
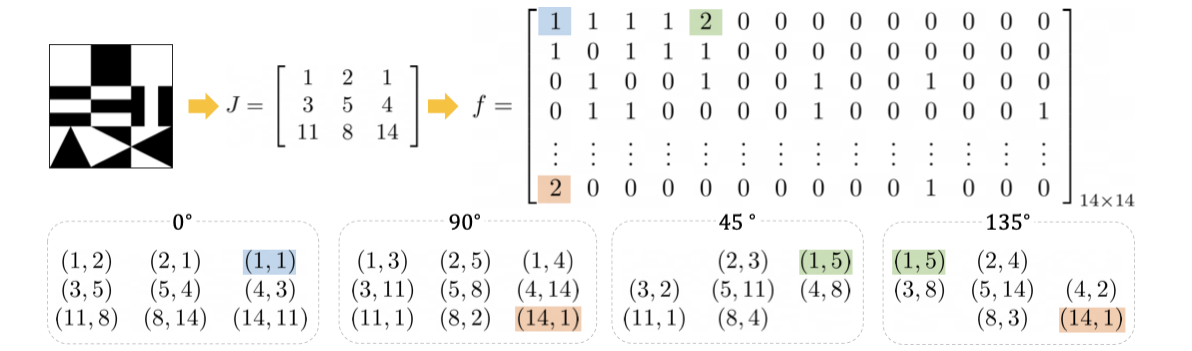
Gray-level co-occurrence matrix computation of a given 3 × 3 Assembly item.

The GLCM was adopted for capturing the configurational disorder (*C_config_*) [39]. Figure 5 illustrates how it is obtained for an Assembly item. First, all geometric shapes used in each item are represented as *J* with the indexes corresponding to each shape defined in Figure 2 and its location. Second, all adjacent pairs along the 0°, 45°, 90°, and 135° directions on *J* are extracted. For example, the second row in *J* is (3, 5, 4), and the ordered pairs along 0°, including the circulant pair, are (3, 5), (5, 4), and (4, 3). Once all pairs are obtained, the number of each pair is imposed on the location in a 14-by-14 matrix *f*, that is, the GLCM, following equation 1. As shown in Figure 5, there are two (1, 5) pairs that correspond to 2 in the (1, 5) coordinate in *f* and one (1, 1) pair that corresponds to 1 in the (1, 1) coordinate. The weighted entropy [40] based on *f* is then calculated using



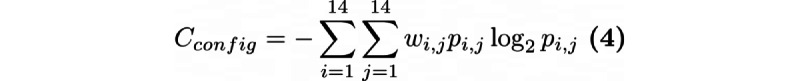



where



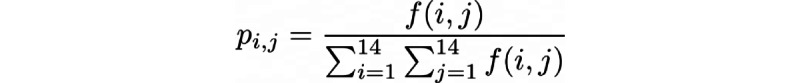



The weight *w_i,j_* estimates the configurational complexity of 2 adjacent elements based on their colors and orientations. To compare the differences among these 14 distinctive shapes, 3 IDs were assigned to each shape to categorize its geometric shape (square, strip, or triangle), color, and orientation (Figure 2). Regarding the IDs, 2 was assigned to the weight if the 2 adjacent shapes had different colors and orientations, and 1 was assigned otherwise.

In Shape-Matching, as the player was asked to find a single shape that best completed the pattern, more shapes used in the pattern do not necessarily indicate greater difficulty in the pattern. Therefore, we only used the configurational complexity to estimate the item difficulty such that *C_play_*=*C_config_*, where *C_config_* is defined as the summation of the weighted entropies based on the 3 GLCMs estimating how frequently a pair occurs horizontally, vertically, and diagonally.

##### Play Complexities of Path-Tracking and Maze

Path-Tracking and Maze do not use the geometric shapes of the cubes and, instead, use a single cube for creating a path. Therefore, the aforementioned method is not applicable. The play complexity of Path-Tracking adopts the network complexity based on the Shannon entropy [41], given by



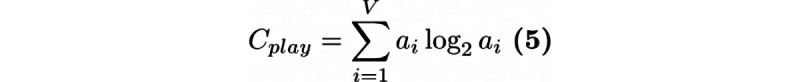



where *V* is the number of vertices and *a_i_* is the associated vertex degree. For Maze, the play complexity is defined as



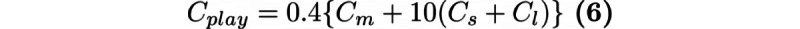



where *C_m_* is the maze complexity using equation 5 and *C_s_* and *C_l_* are calculated using



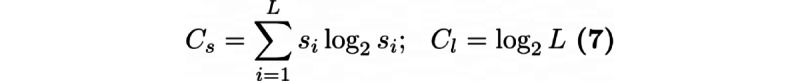



*C_m_* reflects the complexity of the maze itself, but the complexity of solving a maze should also consider the start and end locations. The solution logarithmic complexity (*C_s_*) in equation 7 represents the complexity caused by the vertex degrees, where *L* is the total length of the shortest path solved by the A* algorithm [42] and *s_i_* is the degree of each grid in this solution. The solution length complexity *C_l_* in equation 7 captures the length of the shortest path. In equation 6, the 0.4 value is multiplied to make the complexity values comparable with those of other e-Cube games. In addition, *C_s_* + *C_l_* is multiplied by 10 to balance with the range of *C_m_*.

##### Adaptive Game Generators

The computational measures of play complexity form the basis of the adaptive algorithms, which can automatically generate test items. On the basis of the concept of dynamic difficulty adjustment, we created an adaptive e-Cube system that can adjust the item difficulty based on a player’s performance measured using correctness.

The game begins with an item with a predefined low complexity. If the player answers the first item correctly, it proceeds to the next item with a higher complexity; otherwise, a new item with the same complexity is generated. If 2 consecutive incorrect answers are received, the complexity reverts to the midpoint between the latest correctly answered item complexity and the current incorrectly answered item complexity. The difference between the current and the next complexity value is referred to as a step size that can be either positive, 0, or negative. The game ends at a predefined highest complexity level or when the absolute value of the step size becomes sufficiently small.

**Figure 6:**
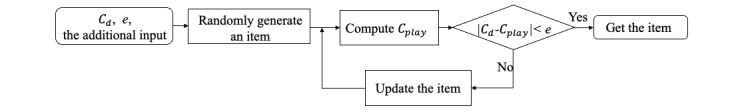
The flowchart of the item generators for Assembly, Sequence-Memory, Spatial-Memory, Path-Tracking, and Maze.

The item generators for all games except for Shape-Matching follow a similar process, shown in Figure 6. The system takes a desired play complexity value *C_d_* and a small tolerance *e* as input and generates a new item with a complexity *C_play_*, where |*C_d_* – *C_play_*|≤*e*. Apart from *C_d_* and *e*, additional input is needed in these games except for Maze. This input is the dimension of the pattern (eg, 2 × 2 or 3 × 3) in Assembly and Spatial-Memory, the number of images to be displayed in Sequence-Memory, and the number of dots to be connected (referred to as nodes) in Path-Tracking. The following steps generate items: (1) the system randomly generates an item based on the inputs; (2) the absolute difference between *C_d_* and *C_play_* is computed, where *C_play_* is the complexity of the current item computed using the proposed measure; (3a) if the absolute difference is smaller than *e*, the system outputs the current item and ends the process; and (3b) if the absolute difference is not smaller than *e*, the system updates one feature of the current item to make the item easier or harder and then goes back to step 2. The features of the item can be geometric shapes in Assembly, Sequence-Memory, and Spatial-Memory; the paths connected by nodes in Path-Tracking; or the position of the end point in Maze.

Shape-Matching uses assembly configurations with embedded patterns where the types of patterns are predefined in the item generator, such as symmetry and rotation. Shape-Matching generates items from a predefined pool. For example, the easiest pattern in the predefined pool is formed by 4 identical shapes, in which one of the shapes will be hidden from players and treated as the missing piece. The item generator for Shape-Matching randomly selects a shape to form the easiest pattern, which leads to different items with the same play complexity.

### Evaluation of e-Cube

The evaluation study focused on the preliminary validation of (1) the proposed play complexity measures that form a basis for developing adaptive games (objective 1) and (2) the preliminary utility and usability of the e-Cube system as an automated cognitive assessment tool (objective 2).

#### Materials and Methods

**Figure 7:**
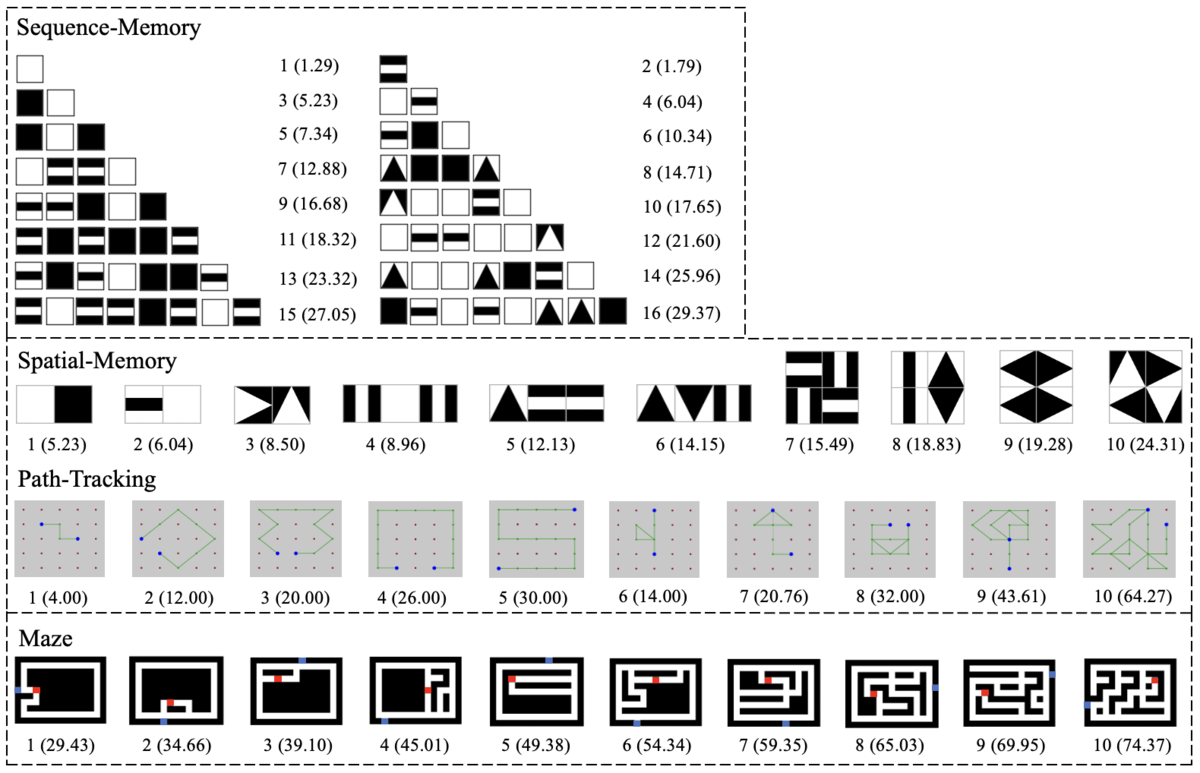
Items with their ordering numbers and play complexity values in the fixed version of Sequence-Memory, Spatial-Memory, Path-Tracking, and Maze.

The study used 2 versions of e-Cube games: e-Cube with the item generators (called *adaptive* e-Cube) and e-Cube with fixed items (called *fixed* e-Cube). Each participant was assigned to 1 of the 2 groups to experience the adaptive or fixed e-Cube games (ie, adaptive group and fixed group). The fixed games provided the same items for each player, whereas the adaptive games offered different items and different numbers of items based on the players’ performance. The fixed versions of Assembly and Shape-Matching used the same items as in the study by Lee et al [26]. The fixed items of the rest of the games are shown in Figure 7.

Objective 1 was tested by performing correlation analyses between the play complexity measures and performance indicators, including the mean correctness and mean completion time obtained by the participants for individual items in the fixed group. For objective 2, the correlations between the raw scores of 3 WAIS-IV subtests (BD, DS, and MR) and the 6 e-Cube games were analyzed to understand their relationships. We also investigated the technical reliability of the system using the false detection rate and usability based on the SUS results.

#### Protocol and Recruitment

This human participant study took place at Texas A&M University (TAMU). Bulk recruitment emails were sent to TAMU communities, and flyers were placed in buildings within the university for recruiting healthy participants aged 18 to 64 years. Once potential participants contacted the research team, a prescreening survey was sent via email to self-identify their eligibility before scheduling a visit. The prescreening survey consisted of 4 questions on age, date of birth, sex, and health conditions. Individuals who were beyond the target age range or had any of the following health conditions were excluded: stroke, other neurological diseases, low vision or blindness with aid, hearing loss or deafness with aid, or difficulties in arm or hand movements for manipulating small objects.

The sample size of a main trial is usually determined through a power analysis, where the variance is known from previous or pilot studies [43]. However, for this preliminary study, we applied the simplest method—sample size rules of thumb, which recommended samples of a minimum of 70 (35 per group) in pilot studies [44,45]. In our study, 80 participants (n=47, 59% male) were recruited and screened. All (80/80, 100%) were eligible and, thus, enrolled in the study. Informed consent and background information (ie, age and sex) were obtained from each participant. Most of the participants were randomly assigned to either the fixed or adaptive group, whereas efforts were made to balance the sex and age distribution between the 2 groups when we placed the participants in the groups toward the end. The fixed group included 48% (38/80) of the participants (23/38, 61% male), and the adaptive group included 52% (42/80) of the participants (24/42, 57% male). Owing to the convenience of recruitment and proximity to the study location, most participants were students from various departments and programs across the TAMU College Station campus, whereas several faculty and staff members, alumni of the university, and a few residents also participated. As a result, 82% (66/80) of the participants were aged between 18 and 30 years, 9% (7/80) were aged between 31 and 40 years, 2% (2/80) were aged between 41 and 50 years, and 6% (5/80) were aged between 51 and 60 years. There were no participants aged >60 years. Age mean, SD, and IQR; age distribution; and sex distribution are summarized in Table 2. We applied a chi-square test at a 95% confidence level to determine if there were differences in sex and age distribution between the 2 groups. The results showed no difference in the proportions of male, female, and intersex participants in the groups (*χ*^2^_2_=0.1, *P*=.76) and no difference in the proportion of age in the groups (*χ*^2^_4_=2.4, *P*=.50).

**Table 2:** Participant demographic data (N=80).

Characteristic			Participants		
			Fixed group (n=38)		Adaptive group (n=42)
Age (years), mean (SD; IQR)			26.71 (9.24; 22.00-28.00)		25.74 (8.50; 20.00-27.25)
**Age range (years), n (%)**					
	18-30	31 (39)		35 (44)	
	31-40	4 (5)		3 (4)	
	41-50	0 (0)		2 (2)	
	51-60	3 (4)		2 (2)	
	>60	0 (0)		0 (0)	
**Sex, n (%)**					
	Male	23 (29)		24 (30)	
	Female	15 (19)		18 (22)	
	Intersex	0 (0)		0 (0)	

The administration order between WAIS-IV and e-Cube was randomized. The order of the 3 subtests of the WAIS-IV followed the standardized protocol (BD, DS, and MR), whereas the order of the 6 e-Cube games was randomized. Upon the completion of both tests, the SUS was administered to each participant. The entire session took approximately 90 minutes: 50 minutes for e-Cube, 25 minutes for the WAIS-IV subtests, 5 minutes for the SUS, and a 10-minute break between e-Cube and the WAIS-IV subtests. Each participant was given a US $10 gift card upon the completion of participation.

#### Scoring System

The e-Cube games have not been standardized yet, and therefore, scoring methods are not finalized at this stage. We benchmarked the scoring methods used for the WAIS-IV subtests and our previous study [26] and modified them to suit the e-Cube games.

The scoring of Assembly considers correctness, item size, and completion time—a 2 × 2 item that is correctly completed within 15 seconds or between 15 and 30 seconds yields 3 or 2 points, respectively; a 3 × 3 item correctly completed within 30 seconds, between 30 and 40 seconds, or between 40 and 60 seconds results in 4, 3, or 2 points, respectively. Shape-Matching, Sequence-Memory, and Spatial-Memory use correctness only as the scoring criteria—2 points for each correct answer and 0 for an incorrect answer. Scoring methods for Path-Tracking and Maze are based on correctness, completion time, and whether the path taken is the shortest. For Path-Tracking, the shortest path finished within 20 seconds, between 20 and 40 seconds, or between 40 and 80 seconds yields 4, 2, or 1 points, respectively; a correct path, but not the shortest, completed within 20 seconds or between 20 and 40 seconds yields 2 or 1 points, respectively. For Maze, the shortest path completed within 10 seconds, between 10 and 20 seconds, or between 20 and 40 seconds yields 4, 2, or 1 points, respectively; a correct path, but not the shortest, completed within 10 seconds or between 10 and 20 seconds yields 2 or 1 points, respectively. Others not satisfying the aforementioned conditions result in 0 points.

The adaptive e-Cube games require some additional considerations for scoring. If an item is generated with the same play complexity as the previous one answered incorrectly, the score for the correct answer is 1 point less than the score used in the fixed version. A total of 2 consecutive incorrect answers result in the system generating an easier item, and in this case, a correct answer for that newly generated item yields only 1 point.

### Statistical Analysis

Correlations were computed to determine the relationships between the computed complexity values and participants’ performance, the connections between the WAIS subtests and the e-Cube games, and the relationships among the 6 e-Cube games. We used the Spearman correlation to measure the monotonic association among them. The correlation is interpreted as “weak,” “moderate,” and “strong/high” when the coefficient is <0.36, between 0.36 and 0.67, and >0.67, respectively [46]. We used 2-tailed *t* tests to identify the mean differences in the game or subtest scores and the SUS scores between the 2 groups.

### Ethics Approval and Informed Consent

This human participant study was reviewed and approved by the TAMU Institutional Review Board (IRB2019-1079D; approval date: December 22, 2020). Informed consent was obtained from all participants before taking part in this study.

## Results

All enrolled participants (80/80, 100%) completed the entire session without withdrawal. The results and findings for objectives 1 and 2 are presented in the following sections.

### Objective 1: Evaluation of the Measures of Play Complexity

The preliminary validity of the proposed play complexity measure (*C_play_*) was evaluated by analyzing the correlations between the *C_play_* values and the performance indicators from the fixed group participants. If the defined complexity measures properly reflected the difficulty associated with the individual items, participants would perform worse on the items with higher complexity values. Two performance indicators were used to evaluate the play complexity measures: (1) mean correctness and (2) mean completion time obtained for each item from the fixed group participants. The correlation analyses were performed at a 95% confidence level between the *C_play_* values and all the mean values. The correlation coefficients *r* with *P* values and 95% CIs are shown in Table 3.

The *C_play_* values showed strong positive correlations with the mean completion time in all e-Cube games, indicating that the items with higher *C_play_* yielded a longer time to answer. Negative correlations between the *C_play_* values and the mean correctness were found in Assembly, Shape-Matching, and Sequence-Memory, indicating that higher *C_play_* items yielded lower accuracies. In Spatial-Memory, we found no substantial correlation between the mean correctness and *C_play_*, mainly because of items 8 and 9 (Figure 7). The symmetry in these items seemed to make them easy to memorize, whereas it was not taken into account for the defined complexity measures. Without these 2 items, a correlation was found as *r*_8_=–0.67 (95% CI –0.93 to 0.066; *P*=.06). A few participants correctly answered all the items in Path-Tracking (12/38, 32%; *P*=.58) and Maze (31/38, 82%; *P*=.07), and therefore, correctness did not yield any significant correlation with *C_play_*.

**Table 3:** Correlations (Spearman r, 2-tailed *P* value, and 95% CIs) between the *C_play_* values and the mean correctness and mean completion time for each item from the fixed group participants.

Game (*df*)		Mean completion time	Mean correctness
**Assembly (20)**			
	*r*	*0.86* ^a^	–*0.50*
	*P* value	*<.001*	*.02*
	95% CI	*0.67 to 0.94*	−*0.46 to 0.43*
**Shape-Matching (10)**			
	*r*	*0.95*	–*0.75*
	*P* value	*<.001*	*.009*
	95% CI	*0.80 to 0.99*	−*0.94 to −0.23*
**Sequence-Memory (16)**			
	*r*	*0.98*	–*0.95*
	*P* value	*<.001*	*<.001*
	95% CI	*0.94 to 0.99*	−*0.98 to −0.86*
**Spatial-Memory (10)**			
	*r*	*0.94*	–0.30
	*P* value	*<.001*	.30
	95% CI	*0.76 to 0.99*	−0.78 to 0.41
**Path-Tracking (10)**			
	*r*	*0.82*	–0.20
	*P* value	*.002*	.58
	95% CI	*0.39 to 0.96*	−0.74 to 0.49
**Maze (10)**			
	*r*	*0.72*	–0.59
	*P* value	*.01*	.07
	95% CI	*0.17 to 0.93*	−0.89 to 0.06

^a^Italics indicate that a correlation existed.

### Objective 2: Evaluation of Preliminary Utility and Usability of e-Cube Games for Cognitive Assessment

#### Overview

The mean, SDs, and IQR values obtained from participants in each group for the WAIS-IV subtests (raw scores) and e-Cube games are summarized in Table 4. We also conducted a 2-tailed *t* test with equal variance (Cronbach α=.05) comparing the test scores from the adaptive and fixed groups to determine whether significant differences existed in mean scores between the 2 groups. The *t* test showed no significant differences in the mean scores of the 3 WAIS subtests—BD (*P*=.37), MR (*P*=.06), and DS (*P*=.18)—between the 2 groups. Group differences were found in Shape-Matching and Sequence-Memory, but not in other e-Cube games.

**Table 4:** Score statistics from the Wechsler Adult Intelligence Scale, Fourth Edition (WAIS-IV), subtests and e-Cube games.

		Fixed group, mean (SD; IQR)	Adaptive group, mean (SD; IQR)	2-tailed *t* test (*df*)	*P* value
**WAIS-IV raw score**					
	BD^a^	50.95 (11,26; 41.00-60.00)	53.02 (9.35; 47.75-60.00)	–0.90 (78)	.37
	DS^b^	28.34 (4.86; 24.75-32.00)	29.98 (5.83; 26.00-34.00)	–1.36 (78)	.18
	MR^c^	21.55 (2.45; 11.00-14.00)	22.62 (2.49; 21.00-24.00)	–1.93 (78)	.06
**e-Cube score**					
	Assembly	54.37 (11.10; 48.75-62.00)	58.52 (12.52; 51.75-67.25)	–1.56 (78)	.12
	Shape-Matching	*16.53 (1.84;* *16.00-18.00)* ^d^	*14.67 (2.86; 13.00-* *16.25)*	*3.42 (78)*	*.001*
	Sequence-Memory	*20.63 (4.63;* *16.00-24.00)*	*17.98 (3.83;* *15.00-20.25)*	*2.81 (78)*	*.006*
	Spatial-Memory	17.21 (2.16; 16.00-18.50)	16.88 (2.93; 15.00-19.00)	0.57 (78)	.57
	Path-Tracking	26.95 (5.83; 24.00-31.00)	26.21 (7.54; 22.00-31.25)	0.48 (78)	.63
	Maze	22.47 (6.27; 18.75-26.25)	22.12 (5.18; 17.00-26.00)	0.28 (78)	.78

^a^BD: Block Design.

^b^DS: Digit Span.

^c^MR: Matrix Reasoning.

^d^Italics indicate that a difference existed.

#### Relationship Between e-Cube Games and WAIS-IV Subtests

We presented the expected relationships between the e-Cube games and the WAIS-IV subtests in Table 1. The evaluation results are shown in Tables 5 and 6, which list the correlations between the e-Cube scores and WAIS-IV subtest scores in the fixed and adaptive groups, respectively. The 2 groups showed somewhat different trends in results. In both groups, Assembly and BD were moderately correlated, as expected in Table 1. Shape-Matching was expected to be correlated with MR, and the results from the adaptive group agreed with this. The Shape-Matching results from the fixed group showed a weak correlation with BD but no correlation with MR. Sequence-Memory and Spatial-Memory were expected to tap working memory as assessed by DS, but only the adaptive version of Spatial-Memory was moderately correlated with DS. The adaptive version of Sequence-Memory and BD also showed a weak correlation. Path-Tracking and Maze were expected to be related to BD and MR, and only the adaptive version of Path-Tracking yielded the expected results. However, no correlations were found between both versions of Maze and any WAIS subtest. Another notable finding was that both versions of Sequence-Memory showed no significant correlation with DS. Overall, the results suggested that the adaptive versions better tap into the cognitive abilities assessed by the 3 WAIS-IV subtests.

We further analyzed the intercorrelations among the e-Cube games (Multimedia Appendix 1 for fixed games and Multimedia Appendix 2 for adaptive games). The adaptive e-Cube showed fewer intercorrelations than the fixed version. In the fixed version, most of the games were somewhat correlated except for Shape-Matching. Both versions of Path-Tracking and Maze were correlated with Assembly.

**Table 5:** Correlations (Spearman r_38_, 2-tailed *P* value, and 95% CIs) between the e-Cube scores and raw scores of the Wechsler Adult Intelligence Scale, Fourth Edition, subtests in the fixed group.

Fixed game			BD^a^		DS^b^		MR^c^
**Assembly**							
	*r*	*0.49* ^d,e^		–0.20		0.10	
	*P* value	*.002* ^d^		.23		.57	
	95% CI	*0.20 to 0.70* ^d^		−0.49 to 0.13		−0.23 to 0.41	
**Shape-Matching**							
	*r*	*0.33*		–0.30		0.09^d^	
	*P* value	*.04*		.07		.57^d^	
	95% CI	*0.01 to 0.59*		−0.57 to 0.02		−0.24 to 0.40^d^	
**Sequence-Memory**							
	*r*	0.31		0.14^d^		0.26	
	*P* value	.06		.40^d^		.11	
	95% CI	−0.01 to 0.57		−0.19 to 0.44^d^		−0.07 to 0.54	
**Spatial-Memory**							
	*r*	0.26		0.23^d^		0.19	
	*P* value	.12		.16^d^		.25	
	95% CI	−0.07 to 0.54		−0.10 to 0.51^d^		−0.14 to 0.48	
**Path-Tracking**							
	*r*	*0.43* ^d^		0.04		0.04^d^	
	*P* value	*.007* ^d^		.81		.80^d^	
	95% CI	*0.13 to 0.66* ^d^		−0.28 to 0.36		−0.28 to 0.36^d^	
**Maze**							
	*r*	0.30^d^		–0.05		0.17^d^	
	*P* value	.06^d^		.78		.29^d^	
	95% CI	−0.02 to 0.57^d^		−0.36 to 0.27		−0.16 to 0.46^d^	

^a^BD: Block Design.

^b^DS: Digit Span.

^c^MR: Matrix Reasoning.

^d^Indicates that the 2 were expected to be correlated in Table 1.

^e^Italics indicate that a correlation existed.

**Table 6:** Correlations (Spearman r_42_, 2-tailed *P* value, and 95% CI) between the e-Cube scores and raw scores of the Wechsler Adult Intelligence Scale, Fourth Edition, subtests in the adaptive group.

Adaptive game			BD^a^		DS^b^		MR^c^
**Assembly**							
	*r*	*0.49* ^d,e^		0.18		0.31	
	*P* value	*<.001* ^d^		.25		.05	
	95% CI	*0.21 to 0.70* ^d^		−0.14 to 0.47		−0.00 to 0.57	
**Shape-Matching**							
	*r*	0.26		0.22		*0.34* ^d^	
	*P* value	.09		.16		*.03* ^d^	
	95% CI	−0.06 to 0.53		−0.10 to 0.50		*0.03 to 0.59* ^d^	
**Sequence-Memory**							
	*r*	*0.34*		0.21^d^		0.11	
	*P* value	*.03*		.19^d^		.50	
	95% CI	*0.03 to 0.59*		−0.11 to 0.49^d^		−0.21 to 0.41	
**Spatial-Memory**							
	*r*	0.09		*0.51* ^d^		0.17	
	*P* value	.59		*<.001* ^d^		.29	
	95% CI	−0.23 to 0.39		*0.24 to 0.71* ^d^		−0.15 to 0.46	
**Path-Tracking**							
	*r*	*0.45* ^d^		–0.01		*0.45* ^d^	
	*P* value	*.003* ^d^		.93		*.003* ^d^	
	95% CI	*0.16 to 0.67* ^d^		−0.32 to 0.30		*0.16 to 0.67* ^d^	
**Maze**							
	*r*	0.30^d^		0.07		0.11^d^	
	*P* value	.05^d^		.62		.49^d^	
	95% CI	−0.01 to 0.56^d^		−0.25 to 0.37		−0.21 to 0.41^d^	

^a^BD: Block Design.

^b^DS: Digit Span.

^c^MR: Matrix Reasoning.

^d^Indicates that the 2 were expected to be correlated in Table 1.

^e^Italics indicate that a correlation existed.

#### Technical Reliability and Usability of e-Cube

The e-Cube technology operated smoothly without any substantial technical issues identified during the study. The false detection rate, defined as the percentage ratio of incorrect detections to the total number of detections, was approximately 0.1% (6/5990). We note that all the analyses and computations mentioned previously were based on the corrected data. To analyze the results of the SUS, the scores from the 10 items were converted into a scale of 0 to 100 [47]. The overall mean SUS score was 83.40 (SD 11.52). There was a significant group difference in the results. The mean of the SUS scores from the fixed group participants was 80.79 (SD 13.23), whereas the mean score from the adaptive group participants was 86.01 (SD 8.75). The 2-sample, 2-tailed *t* test with Cronbach α=.05 showed t_78_=–2.10 (*P*=.04). The result from the adaptive group showed a significantly higher mean SUS score with a smaller SD than the fixed group. On the basis of the industry standard [48], the usability of both fixed and adaptive e-Cube games is considered grade A (ie, the games are acceptable).

## Discussion

### Principal Findings

We presented the design, development, and evaluation of the e-Cube system for automated cognitive assessment. e-Cube is a vision-based system converted from TAG-Games, a computerized system using a set of highly instrumented blocks [28]. e-Cube adopted a set of plastic cubes and a webcam instead, costing only approximately US $50. e-Cube also reduced the labor burden by generating adaptive items, detecting answers and behavior, and scoring autonomously. A total of 6 games—Assembly, Shape-Matching, Sequence-Memory, Spatial-Memory, Path-Tracking, and Maze—were designed using the proposed play complexity measures and adaptive item generators. The e-Cube technology and the adaptive games were evaluated by testing the 2 objectives. This human participant study was conducted on the TAMU campus, and thus, most of our study participants (66/80, 82%) were college students aged between 18 and 30 years, with only 18% (14/80) aged between 31 and 60 years. Therefore, the results must be interpreted considering the skewed age distribution and demographic characteristics.

*Objective 1* was supported by the correlation analyses performed between the *C_play_* values and the 2 performance indicators—the mean correctness and mean completion time obtained from the fixed group. We found that each game was correlated with at least one performance indicator. The *C_play_* values of Assembly, Shape-Matching, and Sequence-Memory showed high correlations with both means. No correlations were found using the mean correctness in Spatial-Memory, Path-Tracking, and Maze. As discussed previously, correctness was not the dominant factor that widened the performance difference in Path-Tracking and Maze; thus, no correlations with correctness were found. For Spatial-Memory, 2 items involved symmetric arrangements of the geometric shapes—which made them easy to memorize regardless of the geometric complexity of the shapes. We used the same play complexity measure for both Spatial-Memory and Assembly, which appeared not to ideally reflect the difficulty associated with such memory tasks despite a high correlation in the mean completion time. This problem can be avoided at the software level by adjusting the algorithm for the item generator. Nevertheless, such symmetric images were rarely created in the adaptive version and, thus, are expected to have minimal effect on the assessment outcome.

To test the preliminary utility of the adaptive e-Cube games for cognitive assessment (*objective 2*), correlation analyses were performed between the scores from the e-Cube games and the WAIS-IV subtests. The adaptive games yielded more significant correlations with the WAIS-IV subtests than the fixed ones. This implies the potential utility of the adaptive feature of the e-Cube games based on the complexity measures. The adaptive version used a discontinuation rule (ie, the substantially small step size leading to the termination of the game), which possibly reduced the number of items in each game, fatigue, and unintended correct answers. For example, given a fixed number of items sorted by increasing difficulties, one may fail to answer correctly in the early items but can unintentionally provide correct answers in the later items. The automatic item generator in the adaptive games adjusts the item complexity based on real-time performance, enabling the system to generate a more appropriate assessment for everyone. Note that the administration of the WAIS-IV also applies the discontinuation rule in the subtests to minimize time [49]. The subtest is terminated when a participant fails to answer a certain number of consecutive items, which differs for each subtest. Intercorrelation analyses also showed that the games in the adaptive version were more independent of one another.

There were some other notable findings from the *objective 2* evaluation study. The mean scores of Shape-Matching and Sequence-Memory in the fixed group were higher than those in the adaptive group (Table 4). In the fixed group, we found that most of the participants (26/38, 68%) correctly answered items 1 to 7 and 9, whereas only 50% (19/38) answered item 8 correctly and 24% (9/38) answered item 10 correctly in Shape-Matching. This inconsistency resulted in a higher mean score in the fixed group, but the results were not correlated with MR. In contrast, the adaptive group showed a significant correlation between Shape-Matching and MR. Sequence-Memory and Spatial-Memory were evaluated to understand which game has a monotonic relationship with DS, but a correlation was only found between the adaptive version of Spatial-Memory and DS. DS measures verbal working memory, which relies on auditory recall of numbers, sequences, and orders. However, Sequence-Memory was performed through the visual recall of geometric images, and Spatial-Memory used visual-spatial images. This fundamental design difference may have led to a lack of correlation. In addition, the language differences in participants and how they differently affect DS scores were not analyzed in this study as we did not collect such background data. Some nonnative speakers mentioned slight difficulty in memorizing the numbers said in English during the DS subtest. This feedback was collected only informally. The Path-Tracking and Maze games were correlated with Assembly, implying that their game settings or measured cognitive outcomes were similar to those of Assembly. Furthermore, Maze was not correlated with any WAIS subtest.

We further analyzed the correlation between the composite scores of the 6 e-Cube games and those of the 3 WAIS subtests. The results were *r*_38_=0.51 (95% CI 0.23-0.71; *P*=.001) for the fixed group and *r*_42_=0.53 (95% CI 0.26-0.72; *P*<.001) for the adaptive group. When only 4 e-Cube games (ie, Assembly, Shape-Matching, Sequence-Memory, and Spatial-Memory) were considered, the results were *r*_38_=0.50 (95% CI 0.21-0.71; *P*=.001) for the fixed group and *r*_42_=0.59 (95% CI 0.34-0.76; *P*<.001) for the adaptive group. Path-Tracking and Maze did not result in any meaningful relationship with the WAIS, and thus, their potential utility in cognitive assessment requires further exploration.

The low false detection rate (0.1%) demonstrated the technical functionality of the e-Cube system. Regarding the usability evaluation (*objective 2*), the average SUS scores from participants in both the fixed and adaptive groups were acceptable based on the industry standard [48]. The adaptive games resulted in a considerably higher mean SUS score with a smaller SD than that of the fixed games. To understand the feedback for individual items, we combined the results from both groups and computed the average rate for each item. The results for the individual SUS items were uniformly positive. For the 5 even-numbered questions that were in a negative tone, such as “I found the e-Cube games unnecessarily complex,” all rates were between 1 (*strongly disagree*) and 2 (*disagree*). For the odd-numbered questions that were in a positive tone, the rates were between 4 (*agree*) and 5 (*strongly agree*) except for the following question—“I think that I would like to use the e-Cube games frequently”—with an average rate of 3.8. This was mainly due to the e-Cube games taking relatively long (approximately 50 minutes) to complete at this preliminary stage. Most of our participants (73/80, 91%) were aged <40 years, so using the game frequently to track cognitive decline was unnecessary for them. We received the highest evaluation of 4.5 on the following item: “I found the various functions in the e-Cube games were well integrated among all questions.”

### Limitations

Most participants (66/80, 82%) were TAMU students aged between 18 and 30 years. The data from participants who were young, educated, and motivated do not represent the general population well. This may also explain why none of the participants withdrew from the study. Furthermore, additional demographic information such as education level, socioeconomic status, race, and ethnicity was not collected in this preliminary evaluation study. A larger-scale validation study will be needed to involve participants from various communities with diverse backgrounds.

The administration order of the 6 e-Cube games was randomized to control for an order effect. The test order can influence the test results and bring about different levels of fatigue [50], so a well-developed cognitive assessment usually requires a standardized administration order. Although order and fatigue effects were not found in some standardized tests [50,51], the impact of the administration order of e-Cube on the scores was not investigated.

The WAIS-IV DS includes Forward, Backward, and Sequencing, which measure auditory working memory and attention with information reordering. However, Sequence-Memory and Spatial-Memory rely on visual recall and do not require any manipulation of information. Therefore, DS may not be an ideal choice for validating these 2 games. Another measure, such as Spatial Span Forward in the Wechsler Memory Scale, Fourth Edition, may be selected to compare the results with those of Sequence-Memory and Spatial-Memory in measuring relevant working memory skills.

### Future Work

The e-Cube technology was developed for fully autonomous administration and scoring of cognitive assessment targeting fine motor, hand-eye coordination, cognitive reasoning, and working memory skills. Building on our prior work [26,28], the technology was converted into a much simpler, cheaper, and easy-to-use form, thus showing potential for use in larger-scale research studies. Our long-term objective is for the e-Cube games to serve as a routine self-assessment tool used by individuals who require continuous monitoring of their cognitive health, such as older adults and people with mild cognitive impairment. Once fully established, e-Cube can also be adapted in clinical settings, especially for remote assessment without requiring in-person interactions with an administrator. Our future work will be geared toward this long-term objective.

This extended human participant study will involve diverse participants (eg, age, sex, education, and socioeconomic status) to better represent the general population. In this future evaluation study, the existing instruments for comparison must be revisited and selected to ensure that the measures match the target cognitive domains of individual e-Cube games. Future work will also aim to establish reliability via test-retest evaluation and the validity of self- and remote administration functions via comprehensive and comparative evaluations. The study to understand the user experiences may also be extended by including an additional set of questionnaires to compare traditional instruments and the e-Cube games to gauge their preference if the e-Cube system is proven to replace some of these. To further improve the technical performance, additional vision processing methods may be added to improve this rate, such as hand detection algorithms to prevent hand motions from interfering with block detection.

The rich data from the e-Cube games on patterns, speed, and characteristics of physical movements applied to the cubes can also be explored to further explicate individual differences and cognitive and fine motor deficits. Such behavioral data may hold important information about individuals, especially those with cognitive deficits exacerbated by fine motor deficits or other behavioral symptoms such as hand tremors. Furthermore, the data provided by e-Cube have the potential to assess one’s cognitive skills in a more objective way than in standard clinical settings. Upon validating its utility as a cognitive assessment tool, our future research may explore the e-Cube games for screening of early signs of neurological diseases. For the e-Cube games to be used as a routine assessment tool, we will consider shortening and gamifying the assessment to make it more fun and engaging. Enhanced graphics and sound and visual feedback mechanisms may be added to the game design. For example, we may benchmark the features of Music Blocks and iSIG-Blocks [52,53], allowing the users to customize audio, tactile, and visual sensory feedback during the cognitive assessment. The current system runs on a low-end laptop with a webcam, whereas further developments can make the algorithms executable on a tablet or cell phone using their built-in cameras. This may further reduce the cost, make it suitable for self- or remote assessment, and support long-term adoption and broader use of the technology.

## Appendix

Multimedia Appendix 1 Intercorrelations (Spearman r_38_, 2-tailed *P* value, and 95% CIs) within the scores of fixed games.

Multimedia Appendix 2 Intercorrelations (Spearman r_42_, 2-tailed *P* value, and 95% CIs) within the scores of adaptive games.

## Abbreviations

BD: Block Design
DS: Digit Span
GLCM: gray-level co-occurrence matrix
MR: Matrix Reasoning
SUS: System Usability Scale
TAMU: Texas A&M University
WAIS: Wechsler Adult Intelligence Scale
WAIS-IV: Wechsler Adult Intelligence Scale, Fourth Edition
