# Supplementary material for: Computerized Block Games for Automated Cognitive Assessment: Development and Evaluation Study
Source: JMIR Serious Games. 2023 May 16;11:e40931. doi: 10.2196/40931 (PMC10230360; doi:10.2196/40931)
Supplement: Multimedia Appendix 1 [file games_v11i1e40931_app1.docx]

**Multimedia Appendix 1.** Intercorrelations (Spearman *r*_38_, 2-tailed *P* value, and 95% CIs) within the scores of fixed games.

| **Fixed Game** | | Assembly | Shape-Matching | Sequence-Memory | Spatial-Memory | Path-Tracking | Maze |
| --- | --- | --- | --- | --- | --- | --- | --- |
| Assembly | | | | | | | |
|  | $r$ | 1 | 0.06 | *0.44*^b^ | 0.21 | *0.49*^b^ | *0.54*^b^ |
|  | $P$ value | −^a^ | .74 | *.006*^b^ | .20 | *.001*^b^ | *<.001*^b^ |
|  | 95% CI | −^a^ | −0.26 to 0.37 | *0.14 to 0.67*^b^ | −0.12 to 0.50 | *0.20 to 0.70*^b^ | *0.27 to 0.73*^b^ |
| Shape-Matching | | | | | | | |
|  | $r$ | 0.006 | 1 | 0.11 | −0.03 | −0.03 | −0.11 |
|  | $P$ value | .74 | −^a^ | .52 | .86 | .88 | .51 |
|  | 95% CI | −0.26 to 0.37 | −^a^ | −0.22 to 0.42 | −0.35 to 0.29 | −0.35 to 0.29 | −0.42 to 0.22 |
| Sequence-Memory | | | | | | | |
|  | $r$ | *0.44*^b^ | 0.11 | $1$ | *0.34*^b^ | *0.35*^b^ | *0.44*^b^ |
|  | $P$ value | *.006* ^b^ | .52 | −^a^ | *.04*^b^ | *.03*^b^ | *.005*^b^ |
|  | 95% CI | *0.14 to 0.67*^b^ | −0.22 to 0.42 | −^a^ | *0.02 to 0.60*^b^ | 0.03 to 0.60^b^ | *0.14 to 0.67*^b^ |
| Spatial-Memory | | | | | | | |
|  | $r$ | 0.21 | −0.03 | *0.34*^b^ | 1 | 0.04 | 0.22 |
|  | $P$ value | .20 | .86 | *.04*^b^ | −^a^ | .79 | .18 |
|  | 95% CI | −0.12 to 0.50 | −0.35 to 0.29 | *0.02 to 0.60*^b^ | −^a^ | −0.28 to 0.36 | −0.11 to 0.50 |
| Path-Tracking | | | | | | | |
|  | $r$ | *0.49*^b^ | −0.03 | *0.35*^b^ | 0.04 | 1 | 0.25 |
|  | $P$ value | *.001*^b^ | .88 | *.03*^b^ | .79 | −^a^ | .13 |
|  | 95% CI | *0.20 to 0.70*^b^ | −0.35 to 0.29 | *0.03 to 0.60*^b^ | −0.28 to 0.36 | −^a^ | −0.08 to 0.53 |
| Maze | | | | | | | |
|  | $r$ | *0.54*^b^ | −0.11 | *0.44*^b^ | 0.22 | 0.25 | 1 |
|  | $P$ value | *<.001*^b^ | .51 | *.005*^b^ | .18 | .13 | −^a^ |
|  | 95% CI | *0.27 to 0.73*^b^ | −0.42 to 0.22 | *0.14 to 0.67*^b^ | −0.11 to 0.50 | −0.08 to 0.53 | −^a^ |

^a^Not applicable.

^b^Italics indicate that a correlation exists.
