# Supplementary material for: Computerized Block Games for Automated Cognitive Assessment: Development and Evaluation Study
Source: JMIR Serious Games. 2023 May 16;11:e40931. doi: 10.2196/40931 (PMC10230360; doi:10.2196/40931)
Supplement: Multimedia Appendix 2 [file games_v11i1e40931_app2.docx]

**Multimedia Appendix 2.** Intercorrelations (Spearman *r*_42_, 2-tailed *P* value, and 95% CIs) within the scores of adaptive games.

| **Adaptive Game** | | Assembly | Shape-Matching | Sequence-Memory | Spatial-Memory | Path-Tracking | Maze |
| --- | --- | --- | --- | --- | --- | --- | --- |
| Assembly | | | | | | | |
|  | $r$ | 1 | 0.20 | 0.12 | 0.17 | *0.58*^b^ | *0.55*^b^ |
|  | $P$ value | −^a^ | .21 | .46 | .27 | *<.001*^b^ | *.001*^b^ |
|  | 95% CI | −^a^ | −0.12 to 0.48 | −0.20 to 0.42 | −0.15 to 0.46 | *0.33 to 0.76*^b^ | *0.29 to 0.74*^b^ |
| Shape-Matching | | | | | | | |
|  | $r$ | 0.20 | 1 | 0.13 | 0.11 | 0.18 | 0.13 |
|  | $P$ value | .21 | −^a^ | .42 | .47 | .24 | .40 |
|  | 95% CI | −0.12 to 0.48 | −^a^ | −0.19 to 0.42 | −0.21 to 0.41 | −0.14 to 0.47 | −0.19 to 0.42 |
| Sequence-Memory | | | | | | | |
|  | $r$ | 0.12 | 0.13 | 1 | 0.21 | 0.20 | 0.16 |
|  | $P$ value | .46 | .42 | −^a^ | .17 | .21 | .30 |
|  | 95% CI | −0.20 to 0.42 | -0.19 to 0.42 | −^a^ | −0.11 to 0.49 | −0.12 to 0.48 | −0.16 to 0.45 |
| Spatial-Memory | | | | | | | |
|  | $r$ | 0.17 | 0.11 | 0.21 | 1 | 0.13 | 0.19 |
|  | $P$ value | .27 | .47 | .17 | −^a^ | .40 | .22 |
|  | 95% CI | −0.15 to 0.46 | −0.21 to 0.41 | −0.11 to 0.49 | −^a^ | −0.19 to 0.42 | −0.13 to 0.47 |
| Path-Tracking | | | | | | | |
|  | $r$ | *0.58*^b^ | 0.18 | 0.20 | 0.13 | 1 | *0.66*^b^ |
|  | $P$ value | *<.001*^b^ | .24 | .21 | .40 | −^a^ | *<.001*^b^ |
|  | 95% CI | *0.33 to 0.76*^b^ | −0.14 to 0.47 | −0.12 to 0.48 | −0.19 to 0.42 | −^a^ | *0.44 to 0.81*^b^ |
| Maze | | | | | | | |
|  | $r$ | *0.55*^b^ | 0.13 | 0.16 | 0.19 | *0.66*^b^ | 1 |
|  | $P$ value | *.001*^b^ | .40 | .30 | .22 | *<.001*^b^ | −^a^ |
|  | 95% CI | *0.29 to 0.74*^b^ | −0.19 to 0.42 | −0.16 to 0.45 | −0.13 to 0.47 | *0.44 to 0.81*^b^ | −^a^ |

^a^Not applicable.

^b^Italics indicate that a correlation exists.
